# On Tailoring Co-Precipitation Synthesis to Maximize Production Yield of Nanocrystalline Wurtzite ZnS

**DOI:** 10.3390/nano11030715

**Published:** 2021-03-12

**Authors:** Radenka Krsmanović Whiffen, Amelia Montone, Loris Pietrelli, Luciano Pilloni

**Affiliations:** 1 ENEA, Materials Technology Division, Casaccia Research Centre, Via Anguillarese 301, 00123 Rome, Italy; radenka.krsmanovic.whiffen@udg.edu.me (R.K.W.); luciano.pilloni@enea.it (L.P.); 2 Faculty of Polytechnics, University of Donja Gorica, Oktoih 1, 81000 Podgorica, Montenegro; 3 Department of Chemistry, Sapienza University of Rome, Piazzale Aldo Moro 5, 00185 Rome, Italy; loris.pietrelli@uniroma1.it

**Keywords:** zinc sulfide, wurtzite, co-precipitation synthesis, solvent recycling, green synthesis, scaling up, pilot plant

## Abstract

Pyroelectric materials can harvest energy from naturally occurring ambient temperature changes, as well as from artificial temperature changes, notably from industrial activity. Wurtzite- based materials have the advantage of being cheap, non-toxic, and offering excellent opto-electrical properties. Due to their non-centrosymmetric nature, all wurtzite crystals have both piezoelectric and pyroelectric properties. Nanocrystalline wurtzite ZnS, being a room temperature stable material, by contrast to its bulk counterpart, is interesting due to its still not well-explored potential in piezoelectric and pyroelectric energy harvesting. An easy synthesis method—a co-precipitation technique—was selected and successfully tailored for nanocrystalline wurtzite ZnS production. ZnS nanopowder with nanoparticles of 3 to 5 nm in size was synthesized in ethyl glycol under medium temperature conditions using ZnCl_2_ and thiourea as the sources of Zn and S, respectively. The purified and dried ZnS nanopowder was characterized by conventional methods (XRD, SEM, TEM, TG and FTIR). Finally, a constructed in-house pilot plant that is able to produce substantial amounts of wurtzite ZnS nanopowder in an environmentally friendly and cost-effective way is introduced and described.

## 1. Introduction

The access to and use of electrical energy contributes significantly to sustainable development in modern societies. One possible way to generate green energy is by harvesting waste heat and converting it into electrical energy, using either thermoelectric or pyroelectric effects. While commercial thermo-electric generators exist today [1], “pyroelectric energy harvesting” is the least well-explored potential energy harvesting technology. Pyroelectric materials operate with a high thermodynamic efficiency and do not require bulky heat sinks as thermoelectric materials do. Some pyroelectric materials are also stable at up to 1200 °C or more, permitting energy harvesting from high temperature sources with increased thermodynamic efficiency. As a result, pyroelectric materials have the potential to harvest energy from naturally occurring ambient temperature changes, and artificial temperature changes due to exhaust gases, and gas or liquid hot flows in industrial processes. Thus “pyroelectric energy harvesting” could be the right methodology to collect at least some of the enormous amount of wasted energy in the form of heat by converting thermal fluctuations into electrical energy (e.g., over half the energy generated from all sources in the U.S. since 2008 was reported lost that way [2]).

Pyroelectric materials are structurally anisotropic solids that exhibit a permanent dipole moment, which is why they can generate electricity from temperature fluctuations. As such, they are promising candidates for energy harvesting at a large scale. Conventional materials of this type are ferroelectrics, mostly oxides with a perovskite structure, like BaTiO3 (BT), PbTiO3 (PT), LiTaO_3_, Lead Magnesium Niobate/Lead Titanate (PMN-PT) or Barium Strontium Titanate (BST) in the forms of single crystals, ceramics or as fillers in polyvinylidene fluoride composite films. Another promising class are non-ferroelectric pyroelectrics: semiconductor materials of wurtzite crystalline structure like CdS, ZnO or ZnS. Due to their non-centrosymmetric nature, all wurtzite crystals have both piezoelectric and pyroelectric properties. Wurtzite-based materials have the advantage of being cheap, non-toxic and offering excellent opto-electrical properties. Their high chemical and thermal stability allow their use at high temperatures in air, whereas ferroelectrics become ineffective when heated beyond their Curie temperature (their T_C_ is usually lower than 150 °C and can increase at nano-scale [3]). In addition, higher thermal conductivity allows wurtzite-based materials to react faster to ambient temperature change. Currently, the application of pyroelectric materials is limited to low-power electronics, to portable systems or tasks needing only μW–mW power [4]; these applications fit well with nanostructured pyroelectric generators that harvest ambient temperature changes and rapidly generate an electrical current in response to those changes [4]. Although pyroelectric materials and thermal energy harvesting have been studied extensively over the last two decades, research on pyroelectric generators is still in the explorative phase. However, some existing systems such as one developed by NASA [5], are able to successfully capture and transform waste heat generated in power plants, jet engines or automobiles. For more powerful, commercially viable generators that are able to capture industry-generated heat, a more solid form is needed, such as ceramics or thin films with improved mechanical strength and greater resistance to thermal shock.

Alongside ZnO, ZnS is one of the most important Cd-free type II-VI semiconductors with excellent optoelectronic and luminescent properties and numerous applications; in particular, nanostructured ZnS is used mainly as a phosphor in optoelectronic and electro-luminescent devices [6,7,8], in catalysis, for solar cells, and lasers [9,10,11] and, being biologically non-toxic, in biomedical labeling [12]. ZnS has two structural forms: a room temperature stable cubic phase (zinc blende or sphalerite, c-ZnS), which at high temperatures (1020 °C for bulk) becomes a metastable, hexagonal wurtzite phase (w-ZnS), lacking structural stability and of limited application. However, a stabilization mechanism for wurtzite is well known: it was found that w-ZnS in nanocrystalline form is a stable material at room temperature [13,14,15,16]. These two phases of ZnS have different valence band structure that manifests as a difference in the bandgap value, with widely accepted (experimental) values of 3.72 eV for sphalerite and 3.77 eV for wurtzite [17].

To the best of our knowledge, w-ZnS has not been studied as a possible energy harvesting pyroelectric material since the early work of Gérard Marchal on w-ZnS thin films [18] despite w-ZnS being isostructural to the well-exploited and widely praised hexagonal ZnO [19]. Reducing production costs for pyroelectric material is the key to marketing an affordable energy harvesting system. In addition, the Tc temperature (1020 °C for bulk material) is high enough for ZnS that it has the ability to operate at higher temperature that is a good match with the working temperature of power plants and automobiles (mostly lower than 200 °C, up to maximum about 400–500 °C). We intend to create ceramics and composite polymer films to be used in pyroelectric harvesters of waste heat coming from either industrial or domestic activities [20].

Only in the last decade has the interest in nanostructured wurtzite ZnS sparked, and numerous different syntheses have been explored, aiming at its controlled production at low temperatures [21]. Cheng et al. [22] prepared w-ZnS nanoparticles by a solvothermal method from homogeneous solutions of Zinc chloride (ZnCl_2_) with S2- as the precipitating anion from thiourea at 180 °C and without a stabilizing agent, while Zhao et al. [23] prepared hexagonal ZnS NPs using ZnCl_2_ and thiourea controlled by tetramethyl ammonium hydroxide in ethyl glycol (EG). A short list of the possible synthesis is reported in Table S1.

We wanted to avoid the use of toxic materials (Tetramethylammonium hydroxide (TMAH), ethylenediamine, and so on) and to use an inexpensive and simple chemical synthesis at a low working temperature. Hence, we investigated the options of creating ZnS nanopowder using a co-precipitation fabrication process, a soft-chemistry approach that is easy to scale-up. The obtained nanopowder has been characterized using TGA, BET, FTIR, TEM and SEM techniques. We provided an insight into how to optimize this synthetic route, given conditions for better purity w-ZnS nanopowder, and provided an outlook on how to expand its production. We built an in-house pilot plant that is able to produce substantial amounts of wurtzite ZnS nanopowder in an environmentally friendly and cost-effective way.

## 2. Materials and Methods

ZnS nanopowder was fabricated using an easy scalable chemical precipitation process. Zn^2+^ ions form a complex with Ethylene glycol (EG) resulting in particle capping upon nucleation. Upon the addition of thiourea (TU) into the preformed Zn-EG complex, a competition between TU and EG (at a high temperature) is performed. We modified the well-known reaction of zinc chloride (Zn^2+^ source) with thiourea (S^2−^ source) dissolved in ethyl glycol (EG) [23] at different molar ratios (R = 0.47–1.22) in medium temperature conditions (140–150 °C) to produce nanocrystalline ZnS of the hexagonal (wurtzite) phase in a series of consecutive experiments. All the chemicals: ZnCl_2_ (Sigma-Aldrich, St. Louis, MO, USA, ≥98%), thiourea (Sigma-Aldrich, St. Louis, MO, USA ≥99.0%), and ethylene glycol (Sigma-Aldrich, St. Louis, MO, USA ≥99.8%), were used as received and without further purification.

Typically, a known quantity of anhydrous ZnCl_2_ and thiourea (CH_4_N_2_S) were dissolved separately in a known volume of EG (the ratio solid:liquid = 1:15) and stirred at 110 °C for 30 min. Subsequently, both mixtures were merged in a larger glass beaker and stirred (300–400 rpm) at a temperature of 140–150 °C for 1–2 h. After the reaction was complete, the white solution was cooled to room temperature. The powders obtained were separated by centrifugation, at 4000 rpm for 5 min, from the solvent and were washed twice with acetone and twice with ethyl alcohol using centrifugation. The washed product was mixed in ethyl alcohol and this solution was dried to powder at 70 °C, in an oven for about 1 h. Following the lab tests, we built an in-house pilot plant able to produce substantial amounts of wurtzite ZnS nanopowder in an environmentally friendly and cost-effective way. To be specific, the pilot plant consists of a 5 L jacketed glass reactor equipped with the automatic control of pH, temperature and mixing speed. The temperature was controlled (±0.01 °C) for the working temperature range 10–200 °C by a thermostat equipped with a programmable temperature fluid control (model Optima TXF200 Heated Circulating Bath).

**Figure 1:**
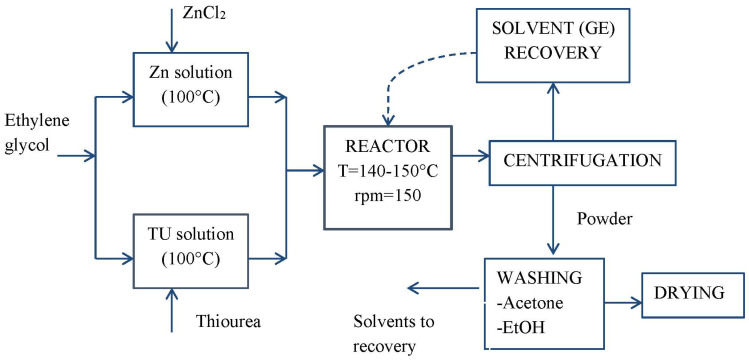
The schematic representation of the ZnS nanopowder synthesis.

The chemical process follows the scheme reported in Figure 1.

The microstructure of the ZnS nanopowders was characterized by X-ray powder diffraction using a SmartLab Rigaku powder diffractometer (Rigaku, Tokio, Japan) equipped with a Cu Kα radiation source and a graphite monochromator in the diffracted beam, operated at 40 kV and 30 mA. The morphology of the samples was investigated by scanning electron microscopy, using a LEO 1530 (Zeiss, Oberkochen, Germany)) instrument. The LEO1530 is a hot cathode field emission SEM equipped with a high-resolution in-lens secondary electron detector, a conventional secondary electron detector, a Centaurus back scattered detector and a XACT microanalysis unit (OXFORD Instruments, Abingdon, United Kingdom) and it was used to provide high-resolution images. TEM images were obtained with a JEOL 2010 TEM (Jeol, Akishima, Japan).

The TG measurements were carried out by a Mettler Toledo thermogravimetric analyzer (Mettler Toledo, Columbus, OH, USA) under a nitrogen atmosphere, where the gas flow was fixed at 20 mL min^−1^. The heating rate was fixed at 5.0 °C min^−1^ and the samples (3–6 mg) were placed in an alumina crucible.

The UV absorption spectra were taken using a Shimadzu UV-1800 Spectrophotometer (Shimadzu, Kyoto, Japan) in the wavelength range 200–850 nm.

The measurement of the specific surface area of the produced wurtzite ZnS powder was carried out using the Brunauer-Emmett-Teller (BET) equation. The samples (about 1 g) were dried in a vacuum system at 120 °C overnight. Nitrogen was used as an adsorbate gas at 77 K (Nova 2200 surface-area analyzer; Quantachrome Instruments, Boynton Beach, FL, USA).

The infrared transmittance measurements of the produced powder were performed using a Thermo Scientific Nicolett 6700 spectrophotometer (Thermo Fisher Scientific, Waltham, MA, USA) at room temperature; the spectrum range was 4000–400 cm^−1^ wavenumber range and a resolution of 2 cm^−1^.

The elemental analysis was performed in order to verify the purity of the powder using a Carlo Erba Instruments EA 1110 CHNS-O elemental analyzer (Egelsbach, Germany).

## 3. Results and Discussion

### 3.1. On Synthesis

In order to produce wurtzite ZnS in an environmentally friendly and cost-effective way, we slightly modified the reaction by employing simple mixing at atmospheric pressure instead of a solvothermal method and, moreover, by recycling the solvent that still contained Zn^2+^ ions. The w-ZnS nanoparticles were synthesized by a co-precipitation reaction using precursor solutions at different Zn^2+^/S^2−^ ratios.

In order to minimize waste according to the circular economy approach in the preparation of high value compounds such as w-ZnS NPs, following the recommendations of the European Environmental Agency [24], the synthesis was designed for reuse with both the reaction media (GE) and the washing solvent.

**Figure 2:**
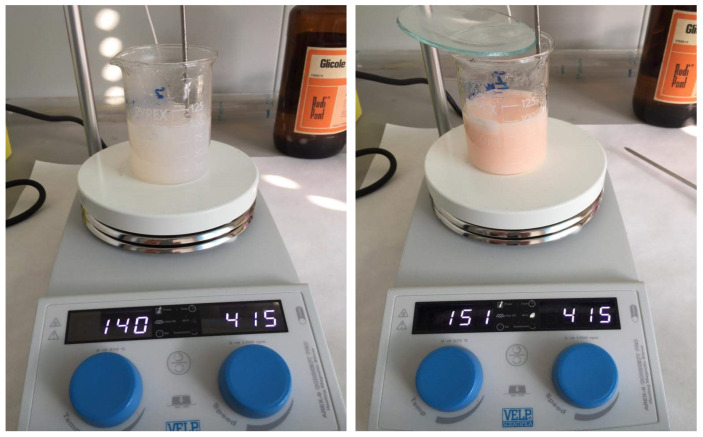
The color of the solution changes with temperature from milky white at 140 °C to rose pink at 150 °C. This change was very quick and easily detectable.

We observed different color formations in the reactive solution during the synthesis process. The starting solution was transparent, and was heated up gradually. At 140 °C, a milky white solution was developed that transformed to rose pink almost instantaneously once the temperature reached 150 °C, as shown in Figure 2.

The pink color observed at about 150 °C is probably due to the formation of a zinc complex favored by the isomerization of thiourea in ammonium thiocyanate. A reversible reaction (1) of thiourea isomerization into thiocyanate (NH_4_SCN) occurring in the range 140–180 °C with an equilibrium ratio of TU:NH_4_SCN at 1:3 [25]. With the increasing temperature (>150 °C), the formation of guanidinium thyocyanate can occur according to reaction (3):

SC(NH_2_)_2_ ↔ NH_4_SCN (1)

SC(NH_2_)_2_ → NH_2_CN + H_2_S (2)

NH_4_SCN + NH_2_CN → [H_2_N^+^=C (NH_2_)_2_] SCN^−^ (3)

The ammonium thiocyanate Formation (1) can promote the precipitation of the tetrathiocyanatozincate(II) anion complex [26].

The experimental results of TU decomposition by TGA indicate that NH_4_SCN, H_2_S, NH_3_, CS_2_, HNCS can be formed [27,28].

**Figure 3:**
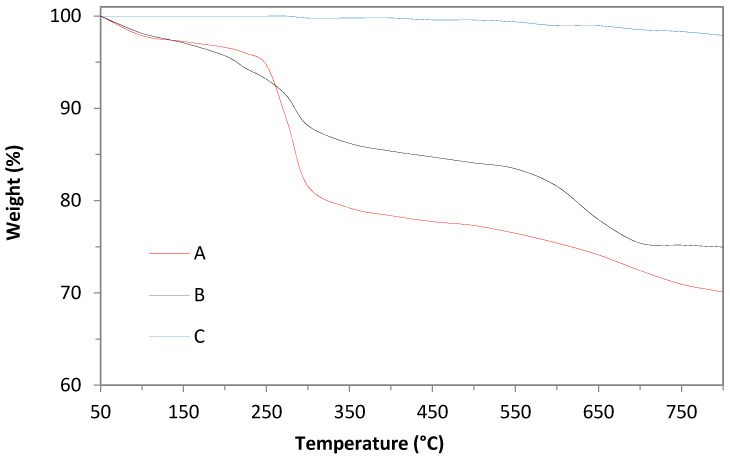
The TGA curves of the thermal decomposition of the produced wurtzite ZnS. Red (A) = washed with water for 5 min, black (B) = washed with acetone and ethanol for 5 min, blue (C) = commercial ZnS powder (UMICORE). The flow rate of N_2_ was 20 mL min^−1^; the heating rate equals 5 °C min^−1^.

Figure 3 presents the TGA curves of the produced w-ZnS; the curves show mass loss in the temperature range 200–330 °C probably due to the production of the carbon disulfide (CS_2_) and ammonia (NH_3_), while at T > 500 °C, we expect other gaseous species such as cyanamide (H_2_NCN), hydrogen cyanide (HCN), carbon dioxide (CO_2_) and carbonyl sulfide (COS) to be formed, as it was reported by Madaraz and Pokol et al. [28].

The complete decomposition of the thio- and cyano- compounds occurs at T > 500 °C, where a weight loss of between 15% and 31% was observed (see Figure 3).

**Figure 4:**
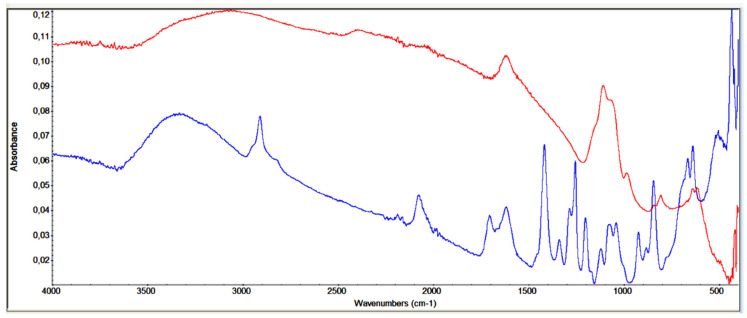
The FTIR spectra of w-ZnS washed solution (acetone and ethanol) for different time: 5 min (blue) and 30 min (red).

The FTIR analysis of w-ZnS powders is shown in Figure 4. The spectrum obtained from the slightly pink colored ZnS powder synthesized at 150 °C show at 1100–1500 nm, the characteristic absorption bands of the sulphur compounds such as C=S, CSNH, SO_2_, SO_2_N, while at 2060 nm, the SCN vibrations and, at 1400 nm, the NH_4_ peak are present. These data confirm the presence of TU degradation compounds as observed by TGA. More research will be conducted to study this degradation mechanism in greater detail [25]. The broad absorption band at 3200 nm can be attributed to OH from the adsorbed H_2_O on the surface of the powder, while the bands at 1560 nm and 1428 nm can be attributed to the zinc carboxylate [29].

On the other hand, the comparison of the UV spectra of the washing solutions (wurtzite nanopowder in washing medium acetone and ethyl alcohol) used for the ZnS nanopowders obtained at 140 °C and 150 °C (Figure S1 in Supplementary Material) showed the presence of a peak (around 320 nm) in the spectrum of the solvent used for the powder obtained at 150 °C, probably attributable to a derivative of the carbodiimide which is one of the products of the decomposition of thiourea.

The elemental analysis shows that after washing the resultant w-ZnS nanopowder twice with acetone and twice with EtOH, impurities are still present. In particular, the powder contains C = 13.77%, N = 1.18% and H = 2.50%. By introducing an additional washing step with cold water, the following elemental analysis was obtained: C = 7.11%, N = 0.34% and H = 1.55%. A successful removal of the solvents and degradation products was achieved by increasing the washing times, as reported in Figure 4.

The effect of the nmZn/nMS molar ratio can be observed in Figure S2 of the Supplementary Material. The quantity of the nanopowder production is strongly correlated to the concentration of zinc ions; in particular, an excess of zinc ions is required to increase the production of w-ZnS nanopowder.

### 3.2. Temperature Effect

Temperature plays an important role in the synthesis of nano w-ZnS. According to Cheng et al. [19], the Zn^2+^ ions and TU homogeneous EG solutions are mixed, and the following reaction can be described:

Zn^2+^ + *n*CS(NH_2_)_2_ → {Zn[CS(NH_2_)_2_]*_n_*}^2+^ (4)

creating a strong coordination complex that at about 110 °C may decompose generating nucleuses and then ZnS crystals having hexagonal form.

Moreover, to avoid obtaining a precipitate containing degradation products of thiourea that are very difficult to wash out, it is necessary that the synthesis reaction take place at 140 °C. We performed dozens of experiments and we can confirm that synthesis at 140 °C is the only way to obtain a white w-ZnS nanopowder as the final product which contains only the solvent (EG) and the reagents (TU and ZnCl_2_) as impurities, which are easy to remove through a standard washing procedure using centrifugation.

**Figure 5:**
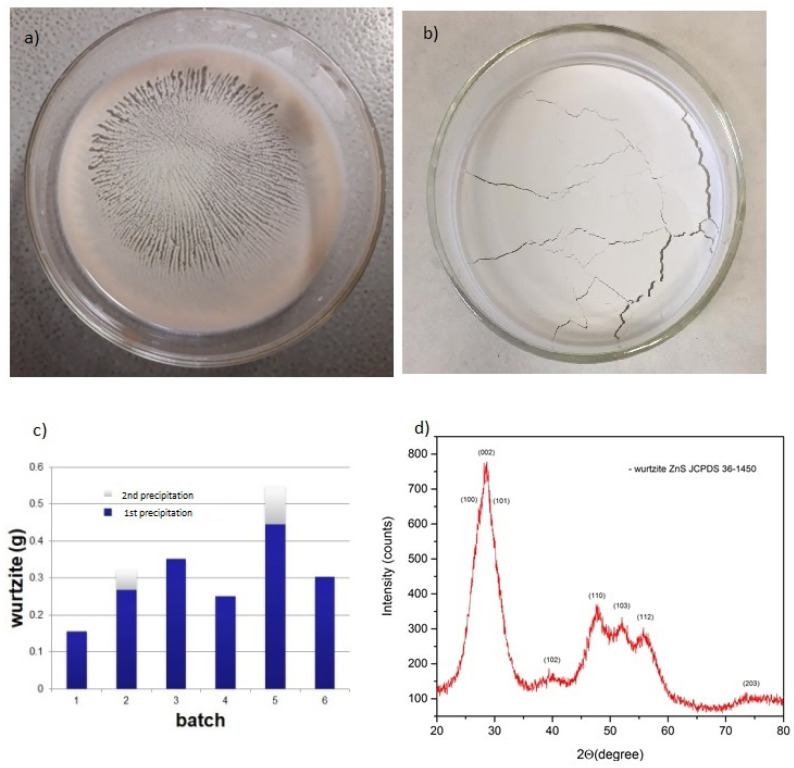
(**a**) Self-alignment of the ZnS wurtzite nanopowder (after thermal treatment at 70 °C for 1 h) into highly ordered arrays; (**b**) The final product—wurtzite ZnS nanopowder; (**c**) The results of the “recycling” synthesis experiments; and (**d**) The XRD diffractogram taken from the produced nanopowder ZnS.

We also investigated options involving tailoring this synthesis route to maximize the production yield of nanocrystalline wurtzite ZnS. We used the same reaction of zinc chloride with thiourea dissolved in ethyl glycol to produce pure, nanocrystalline ZnS of hexagonal phase in a series of consecutive experiments. The amount of the solvent was kept the same (60 mL of ethyl glycol) by re-using the remnants of the solvent from the previous reaction and topping up the quantity lost. The productivity yield increased 3.5 times in 6 successive reactions, from 156 mg to 549 mg per batch at a constant ratio R = mMZn/mMS = 1 (see Figure 5c). The solvent in the last batch solution contained 17 ppm of zinc and 6 ppm of sulphur. From the XRD measurements in Figure S3, we can see that the “standard” sample is more crystalline in respect to the “recycled” ones, as the latter contain more remnants from the organic part, as confirmed by the TGA (Figure 3) and FTIR (Figure 4) measurements.

It is also worth noting that the pilot plant is able to create a considerable amount of nanopowder relatively quickly: across 3 batches produced using the same solvent, (V_EG_ = 600 mL, t = 2 h, T = 140 °C, molar ratio mMZn/mMS ≈ 0.45) 13.732, 11.437, and 12.685 g of wurtzite ZnS were obtained, giving a total of 37.854 g for the whole process.

### 3.3. Structural and Microstructural Characterization

With the synthesis method described above, we were able to produce pure, nanocrystalline ZnS of hexagonal (wurtzite) phase (Figure 5b) in a series of consecutive experiments. After the final drying procedure of the ZnS solution, we observed an unusual phenomenon: a self-alignment of the ZnS wurtzite nanopowder in highly ordered arrays (Figure 5a), probably arising due to the inherent polar nature of the wurtzite nanoparticles and the polar nature of the solvent (ethyl alcohol).

**Figure 6:**
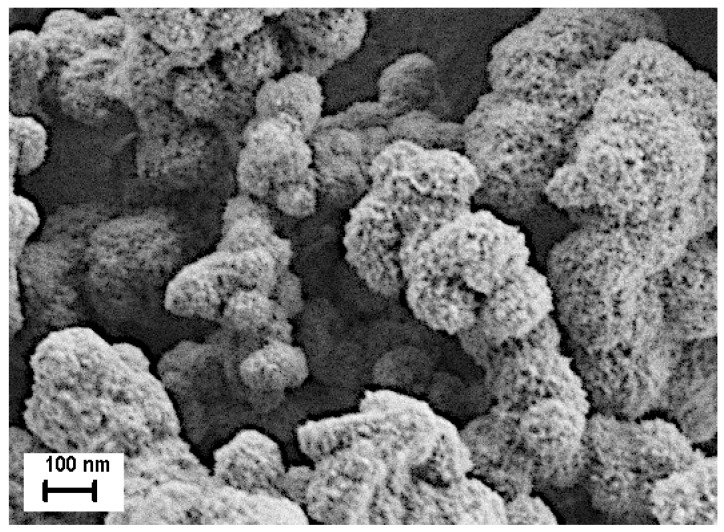
SEM image. The obtained ZnS is constituted by agglomerates composed by nanoparticles whose dimensions are in the range of a few nanometers.

**Figure 7:**
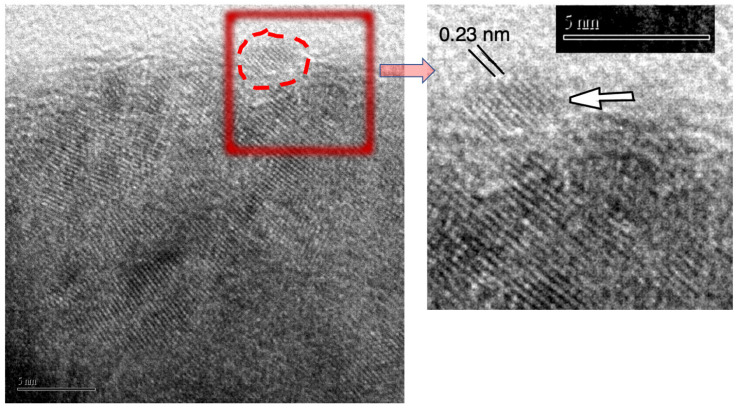
High-resolution TEM image. The images show nanoparticles as small as about 3 nm taken near the surface of an agglomerate. The measured distance between plains is compatible with the distance of (1,0,2) planes of hexagonal ZnS (i.e., about 0.23 nm).

XRD analysis confirmed the hexagonal structure of ZnS (see Figure 5d), while SEM observation (Figure 6) showed nicely agglomerated spheres made of nanoparticles that we could clearly observe in the TEM image (Figure 7). The SEM observations revealed that the nanopowder sample is organized quite uniformly on a large scale in 100–200 nm-size globular structures (see Figure 6). The microstructure at the local level was studied using TEM. HRTEM images taken at higher magnification show that the w-ZnS samples are nanophase materials with crystallite made up of about 3 nm in size. The specific surface area of the ZnS nanopowder was measured as being 38 m^2^/g.

## 4. Conclusions

In order to produce wurtzite in an environmentally friendly and cost-effective way, a co-precipitation reaction including a simple mixing of precursors at atmospheric pressure was explored. In addition, a successful procedure for recycling of the solvent that still contains Zn^2+^ ions (from the previous reaction) was introduced. As a consequence of this greener, “circular approach”, that complements ongoing research trends towards eco-friendly nanoparticle production [30], the productivity yield increased 3.5 times. Following the lab tests, an in-house pilot plant was built, able to produce substantial amounts of wurtzite ZnS nanopowder in an environmentally friendly and cost-effective way, whereby, across three batches prepared in sequence using the same solvent, approximately 38 g were obtained (V_EG_ = 600 mL, t = 2 h, T = 140 °C, mMZn/mMS ≈ 0.45). The yield increased using this molar ratio as reported in Figure S2. Based on the present results, the main advantages of our home-made pilot plant (see Figure S4 of Supplementary Material) include: (i) easy assemblage from commercially available items, (ii) transparency of the glass reactor for clear monitoring of the synthesis process, (iii) production yield of about 18–20% per batch, and (iv) 100% solvent recyclability.

A further investigation regarding the washing procedure of the synthetized w-ZnS nanopowder is envisaged to achieve a higher degree of purity, for example, the use of an ultrasound bath could lead to a greater removal of impurities while reducing the washing time. Our thermogravimetric measurements show that the remnants of ethyl glycol and thiourea decompose completely at 250–290 °C (see Figure 3), hence a flash-heating of the powder might be worth exploring as an additional purification method.

One crucial aspect that highlights the importance of recycling within the chemical process is the solvent recovery and reuse. Our practice allows for a significant reduction in the amount of solvent needed for the w-ZnS nanopowder production process, and we expect to employ the same approach for the synthesis of other similar materials such as ZnO.

## Data Availability

Data is contained within the article or supplementary material.

## Supplementary Materials

The following are available online at https://www.mdpi.com/2079-4991/11/3/715/s1, Table S1: Synthesis of wurtzite ZnS by co-precipitation technique, Figure S1: The UV absorption spectra of the w-ZnS powder washing solutions: w-ZnS produced at 150 °C (blue), at 140 °C (red) and of clean solvent (green), Figure S2: The graph showing the production of w-ZnS (in grams) as a function of the nmZn/nMS molar ratio used in the synthesis, Figure S3: XRD diffractogram taken from the ZnS “standard” samples—red and blue, and from the “recycled” samples—green, orange and purple lines, Figure S4: The glass reactor of the pilot plant and jars containing the recycled solvent (right) and the w-ZnS solution (left). The pilot plant consists of a 5 L transparent jacketed glass reactor with the mechanical stirrer, equipped with a temperature sensor and controller, stirring velocity controller and a pH value indicator, as well as a circulating bath with advanced digital temperature controller.
