# Supplementary material for: On Tailoring Co-Precipitation Synthesis to Maximize Production Yield of Nanocrystalline Wurtzite ZnS
Source: Nanomaterials (Basel). 2021 Mar 12;11(3):715. doi: 10.3390/nano11030715 (PMC8000966; doi:10.3390/nano11030715)
Supplement: Supplementary file 1 [file nanomaterials-11-00715-s001.pdf]

SUPPLEMENTARY INFORMATION

Article

On Tailoring Co-Precipitation Synthesis to Maximize Production Yield of Nanocrystalline Wurtzite ZnS

Radenka Krsmanović Whiffen [1,2], Amelia Montone [1,*], Loris Pietrelli [3] and Luciano Pilloni [1]

[1] ENEA, Materials Technology Division, Casaccia Research Centre, Via Anguillarese 301, 00123 Rome, Italy; radenka.krsmanovic.whiffen@udg.edu.me (R.K.W.); luciano.pilloni@enea.it (L.P.)
[2] Faculty of Polytechnics, University of Donja Gorica, Oktoih 1, 81000 Podgorica, Montenegro
[3] Department of chemistry, Sapienza University of Rome, Piazzale Aldo Moro 5, 00185 Rome, Italy; loris.pietrelli@uniroma1.it
* Correspondence: amelia.montone@enea.it

Table S1. Synthesis of wurtzite ZnS by co-precipitation technique.

Zn precursor	Sprecursor	Coordinating agent	Solvent	Ligand	Synthesis method	Reaction time (h)	Tempera-ture (°C)	Atmo-sphere	Precipi-tation	Phase	Size (nm)	Ref.	Scale-Up
zinc nitrate Zn(NO3)2	thioacetamide		chloroform CHCl3	octylamine	simple mixing of solutions	3	60	AIR	YES	WZ	nanowires (10 × 1.3)	[1]	OK
zinc chloride ZnCl2	thioacetamide		oleylamine	oleylamine	simple mixing of solutions	3	60	AIR	YES	WZ	stacked nanoplatelets	[1]	OK
zinc chloride ZnCl2	thioacetamide		oleylamine	oleylamine & octylamine	simple mixing of solutions	3	60	AIR	YES	WZ	free nanoplatelets	[1]	OK
zinc nitrate Zn(NO3)2	thiourea NH2CSNH2 - (TU)	thiourea (TU)	ethylene glycol (EG)		solvothermal	10	150	N2	YES	WZ	nanoparticles (6)	[2]	OK
zinc chloride ZnCl2	thiourea (TU)	thiourea (TU)	ethylene glycol (EG)		solvothermal	10	150	N2	YES	WZ	nanoparticles (20)	[2]	OK
zinc chloride ZnCl2	thiourea (TU)	ethylene glycol (EG)	TMAH		simple mixing of solutions	2	150–160 (250)	AIR/Ar	YES	WZ	3	[3]	OK
zinc chloride ZnCl2	thiourea (TU)	diethylene glycol			simple mixing of solutions	2	150–160 (250)	AIR/Ar	YES	WZ		[3]	OK
zinc chloride ZnCl2	thiourea (TU)	glycerol			simple mixing of solutions	2	150–160 (250)	AIR/Ar	YES	WZ		[3]	OK
zinc chloride ZnCl2	thiourea (TU)	ethylene glycol (EG)	TMAH		simple mixing of solutions	see supporting info	100–160	AIR	NO	WZ	NPs (5)	[4]	OK

zinc nitrate (ZN)	thiourea (TU)		ethylene glycol (EG)		solvothermal (autoclave)	12	200		YES	WZ	NPs (6)	[5]	
zinc nitrate (ZN)	thiourea (TU)		ethylene glycol (EG)		solvothermal (autoclave)	12	150		YES	WZ	NPs (1.5)	[5]	
zinc nitrate (ZN)	thiourea (TU)		ethylene glycol (EG)		solvothermal (autoclave)	12	180		YES	WZ	NPs (3)	[5]	
zinc nitrate (ZN)	thiourea (TU)		ethylene glycol (EG)		solvothermal (autoclave)	12	230		YES	WZ	NPs (9)	[5]	
zinc acetate	thiourea (TU)	PVP (Mw=58 000)	ethylene glycol (EG)		solution-phase thermal decomposition	3	150	AIR	YES	WZ	spherical NPs (3, 5)	[6]	OK
zinc acetate	thiourea (TU)	tetrabutylammonium hydroxide	ethylene glycol (EG)		microwave-solvothermal process	10 min	140		YES	WZ	nanopowder	[7]	OK
zinc nitrate (ZN)	thiourea (TU)		methanol+benzyl alcohol		solvothermal (autoclave), 10 bars	2	250	N2, autoclave	YES	WZ	layered nanorods (300)	[8]	OK
zinc nitrate hexahydrate	sulfur (nS ≥ nZn)	PEG 400	PEG 400		mild magnetic stirring	3	160		YES	cubic ZB	1D-rods (100)	[9]	OK

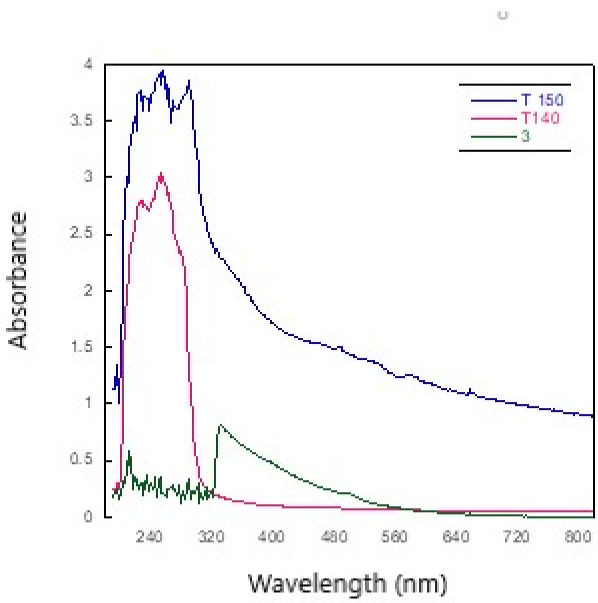

Figure S1. The UV absorption spectra of the w-ZnS powder washing solutions: w-ZnS produced at 150°C (blue), at 140°C (red) and of clean solvent (green).

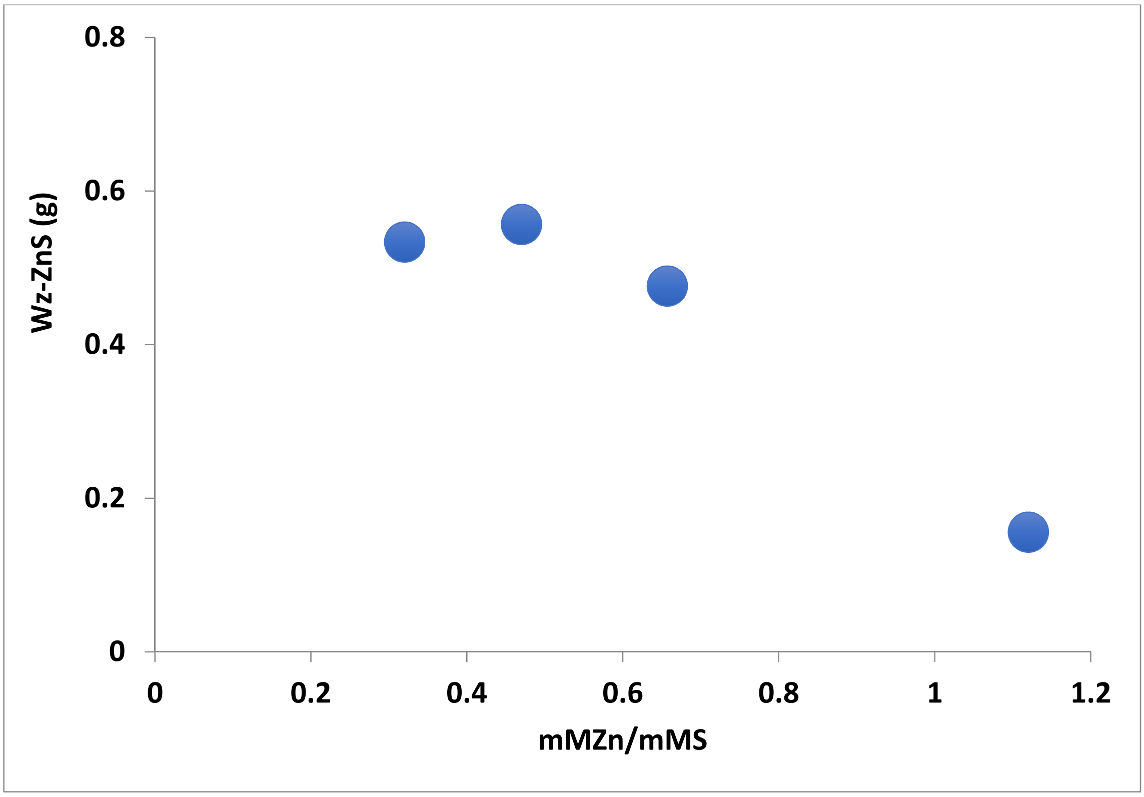

Figure S2. The graph showing the production of w-ZnS (in grammes) as a function of the nmZn/nMS molar ratio used in the synthesis.

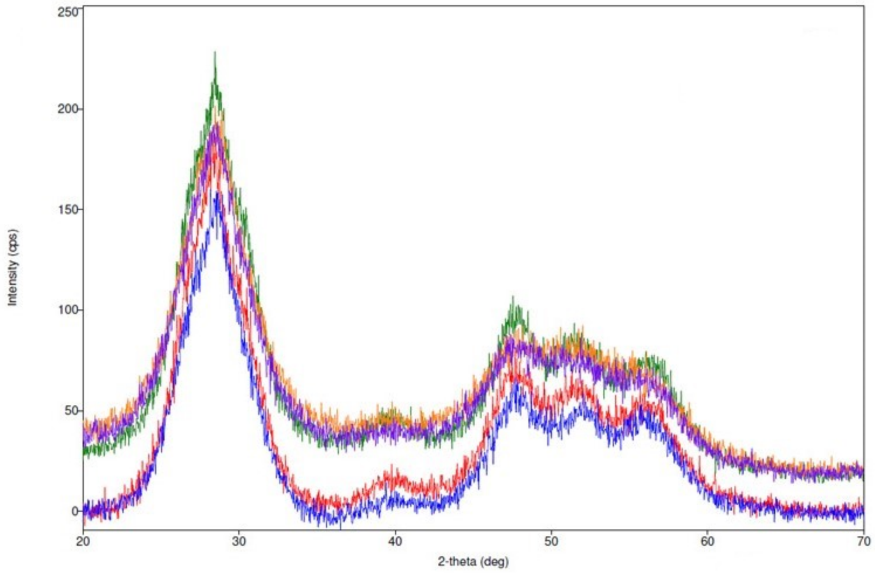

Figure S1. XRD diffractogram taken from the ZnS “standard “samples – red and blue, and from the “recycled” samples – green, orange and purple lines.

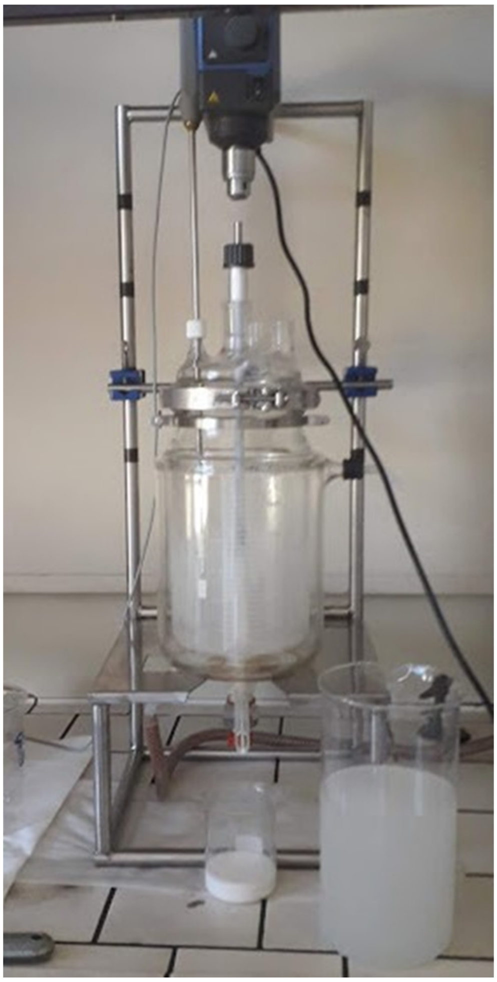

Figure S4. The glass reactor of the pilot plant and jars containing the recycled solvent (right) and the w-ZnS solution (left). The pilot plant consists of a 5 L transparent jacketed glass reactor with the mechanical stirrer,

equipped with a temperature sensor and controller, stirring velocity controller and a pH value indicator, as well as a circulating bath with advanced digital temperature controller.

References:

1. Buffard, A.; Nadal, B.; Heuclin, H.; Patriarche, G.; Dubertret, B. ZnS anisotropic nanocrystals using a one-pot low temperature synthesis. *New J. Chem.* **2015**, *39*, 90–93,
2. Cheng, Y.; Lin, Z.; Lü, H.; Zhang, L.; Yang, B. ZnS nanoparticles well dispersed in ethylene glycol: coordination control synthesis and application as nanocomposite optical coatings. *Nanotechnology* **2014**, *25* 115601
3. Y. Zhao, Y. Zhang, H. Zhu, G. C. Hadjipanayis, J. Q. Xiao, Low-Temperature Synthesis of Hexagonal (Wurtzite) ZnS Nanocrystals. *J. Am. Chem. Soc.* **2004**, *126*, 6874. https://doi.org/10.1021/ja048650g
4. Dawood, F.; Schaak, R.E. ZnO-Templated Synthesis of Wurtzite-Type ZnS and ZnSe Nanoparticles. *J. Am. Chem. Soc.* **2009**, *131*, 424, https://pubs.acs.org/doi/pdfplus/10.1021/ja808455u
5. Biswas, S.; Kar, S. Fabrication of ZnS nanoparticles and nanorods with cubic and hexagonal crystal structures: a simple solvothermal approach. *Nanotechnology* **2008**, 19, 045710
6. Hu, J.S.; Ren, L.L.; Guo, Y.G.; Liang, H.P.; Cao, A.M.; Wan, L.J.; Bai, C.L. Mass Production and High Photocatalytic Activity of ZnS Nanoporous Nanoparticles. *Angewandte Chemie International Edition* **2005**. *44*, 1269–1273
7. La Porta, F.A.; Andrés, J.; Li, M.S.; Sambrano, J.R.; Varela, J.A.; Longo, E. Zinc blende versus wurtzite ZnS nanoparticles: control of the phase and optical properties by tetrabutylammonium hydroxide, *Phys. Chem. Chem. Phys* **2014**, *16*, 20127–20137, https://doi.org/10.1039/C4CP02611J
8. Liu, Y.; Hu, J.; Zhou, T.; Che, R.; Li, J. Self-assembly of layered wurtzite ZnS nanorods/nanowires as highly efficient photocatalysts. *J. Mat. Chem.* **2011**, *214*, 16621–16627.
9. Zhou, D.J.; Xie, X.Y.; Zhang, Y.L.; Guo, D.Y.; Zhou, Y.J.; Xie, J.F. Facile synthesis of ZnS nanorods in PEG and their spectral performance. *Mater. Res. Express* **2016**, *3*, 105023
